# Evaluation of Fine and Ultrafine Particles Proportion in Airborne Dust in an Industrial Area

**DOI:** 10.3390/ijerph18178915

**Published:** 2021-08-25

**Authors:** Ondrej Machaczka, Vitezslav Jirik, Viera Brezinova, Adela Vrtkova, Hana Miturova, Petra Riedlova, Andrea Dalecka, Barbara Hermanova, Hana Slachtova, Grzegorz Siemiatkowski, Leszek Osrodka, Radim J. Sram

**Affiliations:** 1 Centre for Epidemiological Research, Faculty of Medicine, University of Ostrava, 703 00 Ostrava, Czech Republic; ondrej.machaczka@osu.cz (O.M.); Viera.Brezinova@seznam.cz (V.B.); adela.vrtkova@vsb.cz (A.V.); petra.riedlova@osu.cz (P.R.); andrea.dalecka@osu.cz (A.D.); barbara.hermanova@osu.cz (B.H.); hana.slachtova@osu.cz (H.S.); radim.sram@osu.cz (R.J.S.); 2 Department of Epidemiology and Public Health, Faculty of Medicine, University of Ostrava, 703 00 Ostrava, Czech Republic; 3 Department of Applied Mathematics, Faculty of Electrical Engineering and Computer Science, VSB—Technical University of Ostrava, 708 00 Ostrava, Czech Republic; 4 Institute of Public Health in Ostrava, 702 000 Ostrava, Czech Republic; hana.miturova@zuova.cz; 5 Lukasiewicz Research Network—Institute of Ceramics and Building Materials, 31-983 Cracow, Poland; g.siemiatkowski@icimb.pl; 6 Institute of Meteorology and Water Management National Research Institute, 01-673 Warsaw, Poland; leszek.osrodka@imgw.pl

**Keywords:** suspended particulate matter, mass concentration, respirable fraction, fine and ultrafine fraction

## Abstract

The health impacts of suspended particulate matter (SPM) are significantly associated with size—the smaller the aerosol particles, the stronger the biological effect. Quantitative evaluation of fine and ultrafine particles (FP and UFP) is, therefore, an integral part of ongoing epidemiological studies. The mass concentrations of SPM fractions (especially PM_2.5_, PM_1.0_, PM_0.25_) were measured in an industrial area using cascade personal samplers and a gravimetric method, and their mass ratio was determined. The results of PM_2.5_, PM_1.0_ were also compared with the reference measurement at stationary stations. The mean ratios PM_2.5_/SPM, PM_1.0_/SPM, and PM_1.0_/PM_2.5_ were 0.76, 0.65, and 0.86, respectively. Surprisingly, a mass dominance of UFP with an aerodynamic diameter <0.25 μm (PM_0.25_) was found with mean ratios of 0.43, 0.57, 0.67 in SPM, PM_2.5_ and PM_1.0_. The method used showed satisfactory agreement in comparison with reference measurements. The respirable fraction may consist predominantly of UFP. Despite the measures currently being taken to improve air quality, the most biologically efficient UFP can escape and remain in the air. UFP are currently determined primarily as particle number as opposed to the mass concentration used for conventional fractions. This complicates their mutual comparison and determination of individual fraction ratios.

## 1. Introduction

A number of epidemiological studies have shown associations between exposure to suspended particulate matter and sometimes total suspended particulate matter, i.e., all particles surrounded by air in a given volume of air [1], hereinafter SPM, and premature death or an increased incidence of disease [2]. In particular, long-term exposure to SPM increases overall mortality and has negative health effects, especially on the respiratory, cardiovascular, and metabolic systems, but also on cognitive health and early childhood development [3] in association with the respirable fraction (PM_2.5_). New epidemiological studies point to a possible link between PM_2.5_ and the development of dementia, even at relatively low exposure levels [4,5]. Exposure to SPM, especially to so-called fine particles, is thought to increase the risk factors for cognitive decline and dementia in elderly life [5]. Within FP, we can also distinguish so-called extremely fine particles (<1.0 µm, PM_1.0_, EFP) and the finest, so-called ultrafine particles (≤0.1 µm, PM_0.1_, i.e., nanoparticles, hereinafter UFP) [6,7].

Oxidative stress is the most common mechanism of SPM induced adverse health effects [8]. In addition, SPM initiates inflammatory damage and increase of proinflammatory mediators, and many others adverse effects including cellular mutagenicity and DNA damage [8,9]. These effects are stronger for FP, EFP and UFP because of their deeper penetration into the respiratory tract up to the alveoli, and 50% is retained in the lung parenchyma [10]. In general, studies demonstrate that the smaller particles show higher toxicity due to mechanisms of oxidative stress and inflammation. In addition, the smallest particles (UFP) can be translocated from the lungs to the bloodstream with a consequent direct toxic effect [7,9,10].

Within the EU, in terms of air pollution, one of the most polluted areas is the Upper Silesian metropolitan area located on the borders of the northeast of the Czech Republic (CR) and the south of Poland [11]. The main sources of air pollution are industry and power engineering, automobile traffic, and local heating. Deposits of high-quality coal, which were discovered in 1763 in the present territory of the city of Ostrava, resulted in the origin of heavy industry (the establishment of ironworks). Thus, industries such as metallurgical, heavy engineering, chemical, power production, and construction predominate today [12].

Studies currently underway as part of the excellent HAIE (Healthy Aging in Industrial Environment) project conduct research in this area and address the assessment of the effects of selected environmental factors on the health and aging of the population in and outside the industrial region. Part of this research is also the evaluation of the roles of the FP and UFP on a number of health indicators in a person’s aging from birth. This work was created within the HAIE project (https://haie.osu.cz/en/programs/ (accessed on 18 June 2021)).

The main objective of this submitted study was to evaluate and determine the proportion of SPM fractions, i.e., PM_2.5_, PM_1.0_ and PM_0.25_, resp. FP, EFP and UFP in an industrial area. The partial aim was to determine the parameters of the method of measuring FP using personal sampling equipment.

There is an absence of data from government agencies for concentrations of PM1.0 [13]. Some studies mention relatively constant value of 0.75 for the PM_1.0_/PM2.5 ratio [13,14]. This value might be useful for the estimation of PM1.0 where no measured data is available [13]. But for PM_0.1_, there is not much data about proportion in other conventional fractions. Only a few experimental studies have measured PM0.1 or a fraction close to PM1.0 by their mass [15,16].

The use of the present method is unique. It does not copy research carried out at monitoring stationary stations and brings new results (not as particulate number concentration, but as mass concentration) important for the assessment of long-term exposures in relation to the known mass concentrations of PM_10_ and PM_2.5_ fractions.

## 2. Materials and Methods

### 2.1. Material

**Figure 1:**
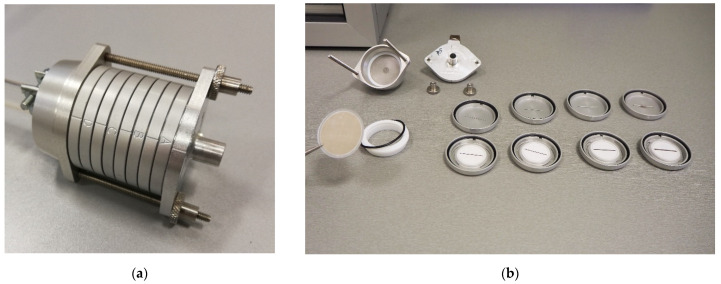
Cascade impactor: (**a**) assembled cascade impactor; (**b**) disassembled cascade impactor—five impact stages with exposed filters. According to the producer https://www.skcinc.com/products/sioutas-five-stage-cascade-impactor (accessed on 13 August 2021) and EPA archive documents https://archive.epa.gov/nrmrl/archive-etv/web/pdf/vr_skcsioutas.pdf accessed on 13 August 2021).

Sampling of SPM fractions was performed with a personal sampling apparatus consisting of a pump (Leland Legacy SKC, constant flow rate of 9 L/min) and a cascade impactor (Sioutas SKC) with PTFE (Polytetrafluoroethylene) filters (hereinafter the impactor). The impactor consisted of four impact stages (four filters with a diameter of 25 mm—pore size 0.5 µm) and an end filter (diameter 37 mm—pore size 2.0 µm), see Figure 1, which allowed the separation and capture of SPM in five filter fractions, see Table 1. A A Defender 500 Mesa Laboratories calibrator was used to calibrate the flow. The individual concentrations captured on the filters were determined gravimetrically.

Total fraction of PM_2.5_ represents the sum of the concentrations of filters B, C, D, E as well as the sum of the concentrations of C, D, E for PM_1.0_, respectively. The sum of the concentrations of all filters indicates the SPM according to the ISO and European Committee for Standardization (EN) standards for air quality and workplace atmospheres [1,17,18,19], see Table 1.

### 2.2. Location of Sampling

**Figure 2:**
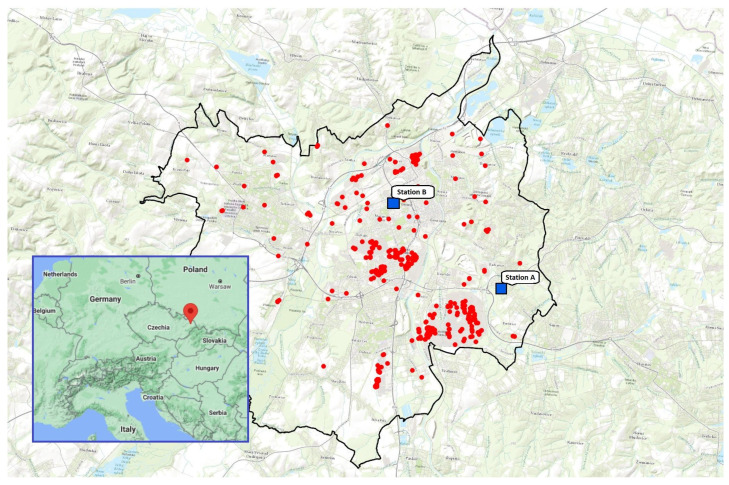
Map of sampling location (city of Ostrava, Czech Republic) with two monitoring stations (Station A and Station B) and with main industry sources of air pollution (marked as circles). Data from Czech Hydrometeorological Institute [20].

The measurement occurred in the city of Ostrava (Figure 2), which is one of the industrial centres of the Upper Silesian region of Europe located near the border of the Czech Republic with Poland. The Ostrava agglomeration is considered one of the most polluted areas in Europe in terms of air pollutants, i.e., SPM [11]. The main sources of air pollution are industry and power engineering, automobile traffic, and local heating. Main industry sources of air pollution are shown in Figure 2. Geographic and meteorological conditions significantly influence the spatial and temporal distribution of air pollutant concentrations. The leading causes of accumulation of air pollutants in this region are meteorological and geological conditions in Upper Silesian Basin, the concentration of industry and solid fuel home heating [12].

### 2.3. Procedure for Determining the Proportion of Individual Fractions

The fractions of SPM were measured by using an impactor (impactor measurement, IM). Two different measurement strategies were conducted. First, the stationary measurements were performed in two localities of Ostrava (see the Section 2.4). Second, personal measurement of the participants recruited into the HAIE project was carried out during their all-day normal activities, including sleeping. Personal measurement was performed from September 2019 to November 2020 (inclusively). Prior to the actual measurement, the pure PTFE filters were conditioned (see the Section 2.4) in a desiccator and weighed on an analytical balance. After 24 h of sampling (13 m^3^ of air collected), the filters were reconditioned under the same conditions as before sampling and weighed on the same analytical balance.

### 2.4. Procedure for Determining the Parameters of the Impactor Measurement

The data from two air pollution monitoring stations (reference measurement, RM) from Ostrava city were used to make a comparison with the impactor measurement (IM). The monitoring station was located according to the Directive 2008/50/EC of the European Parliament and of the Council (see Figure 2). Both stations belong to the Regional Institute of Public Health (RIPH), a medical facility set up by the Ministry of Health, providing a wide range of health and laboratory services. Both stations are operated by the RIHP laboratory accredited according to the ISO and International Electrotechnical Commission (IEC) standard [21] and provide continuous measurement of PM_2.5_ and PM_1.0_. The first station (station A) is a stationary station, which is included in the nationally verified air quality database. The second (station B) is operated under the same conditions for local-regional purposes. A GRIMM 180 analyser was used in both stations for continuous measurement of PM_2.5_ and PM_1.0_ fractions. The analyser works on the principle of light scattering, where a laser diode serves as a light source. The air sample is taken by a sampling head located between a height of 1.5 m and 4 m above the ground.

The impactors were placed on the outer shell of both stations, which was anchored and secured to adverse external influences that could affect the measurement results (e.g., rain, snow, etc.). In the case of station A, the impactor was taken out on the roof, and in the case of station B it was anchored to the side shell. Measurements conducted to determine the parameters of the IM method were performed in the winter period from 12 February to 1 March and at the turn of spring/summer (hereinafter the summer period) from 29 April to 27 June. During the given period, seven 24-hour measurements were performed in each locality (covering all days of the week), and two 48-hour measurements. In winter, in case of frost (below −5 °C) measurements did not occur. Clean and exposed filters were always conditioned for at least 24 h in a medium of approx. RH 43%/25 °C in a desiccator with a saturated K_2_CO_3_ solution before weighing. After conditioning, the filters were weighed on an analytical balance (weighing 10 μg). Selected clean and exposed filters were weighed repeatedly to determine some parameters of the method.

#### IM Method Accuracy Estimation

The estimation of accuracy (i.e., systematic error) was performed by determining the average difference between the IM results and the results obtained from stationary stations A and B, which were considered as references (RM). The precision was assessed by estimating the standard deviation (sd) or coefficient of variance (CV) of multiple (repeated) weighing of selected exposed filters. The detection limit was calculated by estimating the standard deviation of repeated determinations of blanks (pure filters).

### 2.5. Statistical Analysis

Primarily, compositional data analysis was used to investigate the filter fraction com-positions of the collected samples [22], and tools of descriptive analysis of compositional data were applied, such as the estimation of compositional mean and variation matrix [23]. For comparison of the compositions of different fractions in seasons, the James test was used. For further description, ternary diagrams were constructed. Nevertheless, the sample characteristics (minimum, quantiles, maximum, etc.) of the original dataset are also provided.

Secondly, we used the original data to test the agreement between RM and the IM of fraction concentrations. The data were visualized with correlograms and Bland-Altman plots. The differences of the two measurement methods were calculated and analyzed with methods of statistical inference, with the level of significance set to 0.05. The reliability of impactor measurements was evaluated with the intraclass correlation coefficient. Statistical analysis was performed in R software (version 3.6.2, R Core Team, Vienna, Austria).

## 3. Results

### 3.1. Filter Fraction Analysis

A total number of 75 samples (375 measurements) were processed to determine the proportion of individual SPM fractions. Sample characteristics of the original dataset (measured concentrations) and analysis of SPM fraction composition are included in Table 2 and Table 3. For SPM fraction composition, analysis of the transformation of the variation matrix was used. A relatively stable proportionality between the filter fractions was found. For further calculations, data adjusted according to the detection limit were used, i.e., if a value below the detection limit was measured, it was replaced by half the value of the given detection limit (see Table 6).

Table 4 shows a characterization of the mean composition of filter fractions. A significant ratio of filter E particles (UFP) in SPM fractions is evident from these, and a significant difference between evaluated seasons was found (*p* value = 0.032).

**Figure 3:**
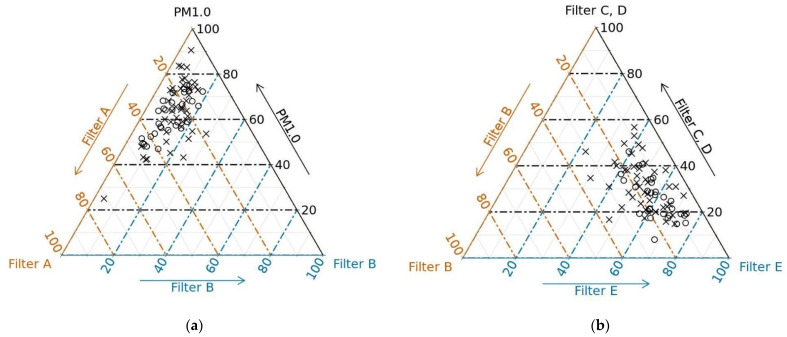
Ternary diagrams. The shape of the points corresponds with seasons (circle = spring, summer, cross = autumn, winter). (**a**) Ternary diagram—filter A, filter B and PM_1.0_ (sum of all filter represents SPM; (**b**) ternary diagram—filter B, filter C + D and filter E (sum of filter B + C + D + E represents PM_2.5_).

In Figure 3, ternary diagrams of the selected filter fractions are shown. The ternary diagram (a) shows the relation between filter fractions A, B and the fraction PM_1.0_ (sum of filter fractions C, D, E), which together represent the fraction SPM. The ternary diagram (b) shows the relation among filter fractions B, C + D, E, which together represent the fraction PM_2.5_. From these graphic representations, the predominance of PM_1.0_ in SPM and filter fraction E (particles < 0.25 µm, ultrafine fraction) in PM_2.5_ is evident.

### 3.2. Determination of IM Method Parameters

#### 3.2.1. Accuracy Estimation

**Figure 4:**
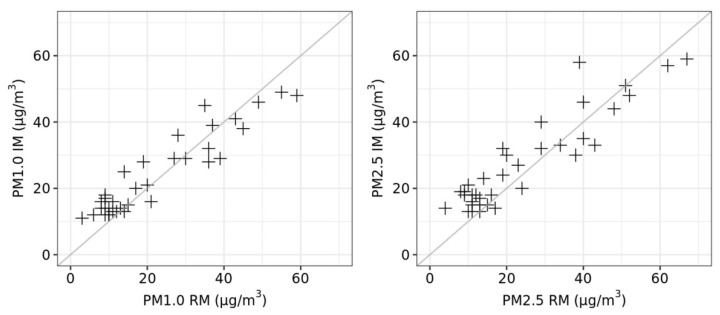
Correlograms. Analysis of agreement between RM and IM. The grey line indicates perfect agreement in measured concentrations.

**Figure 5:**
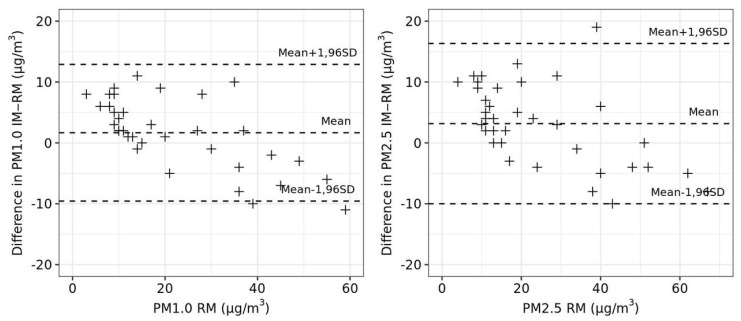
Bland-Altman plots of PM_1.0_ and PM_2.5_ (RM) against the difference in PM_1.0_ and PM_2.5_ (IM minus RM) with the limits of agreement.

The summary values obtained from stations A and B are denoted as RM (reference measurements) and the compared values as IM (impactor measurements). Figure 4 and Figure 5 show a graphical analysis of the agreement between measurement methods RM and IM. Correlograms in Figure 4 and Bland-Altman plots in Figure 5 suggest satisfactory agreement of RM and IM. Table 5 shows the analysis of differences in measured concentrations (IM minus RM). In case of good agreement, the mean of differences should be close to zero, and the paired t-test for the significance of the mean of differences should indicate an absence of significance (i.e., *p*-value ≥ 0.05). Furthermore, an intraclass correlation coefficient close to 1 is desirable. More detailed analysis by season suggested that higher and significant differences in measurements were observed in summer.

#### 3.2.2. Precision, Expanded Uncertainty and Detection Limit

Table 6 contains the precision, expanded uncertainty, and detection limits of gravimetric determination of airborne aerosols from measurements by IM. Precision was calculated from repeated measurements of exposed filters (*n* = 375) and expressed by the mean coefficient of variance (CV) in percentages. Extended uncertainty of the method was calculated from the coefficient of variance as 2*CV according to the EN standard [24]. Detection limit was calculated from repeated measurements of blanks (*n* = 110).

## 4. Discussion

The results of SPM fraction analysis (Table 4) show that the mean ratio PM_2.5_/SPM, PM_1.0_/SPM, and PM_1.0_/PM_2.5_ was 0.76, 0.65 and 0.86, respectively. Relatively stable proportionality between the filter fractions was observed in our samples (Table 3). In general, the proportionality depended on the aerosol composition, which varied with the meteorological conditions by changing the proportion of individual aerosol sources.

Tronville and Rivers in their work [13] reviewed eight studies and obtained a value of 0.75 for the ratio of PM_1.0_/PM_2.5_ as an average value for the purpose of mathematical modeling. They stated that this value is reasonably constant and might be useful for the estimation of PM_1.0_ where no measured data is available [13]. A ratio 0.75 is consistent with the results found, for example, in the Polish study [14] that took measurements in the cross-border Upper Silesian Region near four actively working coal power plants and four coking plants. In comparison, the mean ratio PM_1.0_/PM_2.5_ of 0.86 demonstrated in our work is higher. The results of the Italian study [15], occurring in the city center of Padova, also mentioned a higher ratio (0.96). In this study personal cascade impactor samplers were used for concentration measurement, but ion chromatographic analysis was used. The higher ratio in the Italian study could be due to the sampling campaigns, which were conducted only in the winter season, compared to spring and summer seasons (out of the heating season) in the Polish study. As can be seen from the results of different studies, the contribution of PM_1.0_ to PM_2.5_ had a large variability. This could be caused, for example, by diurnal and seasonal periodicity, proximity to land or sea, locations, altitude, and latitude [25].

When comparing the ratios of SPM fractions from our measurements with respect to the seasons (Table 4), slightly higher average ratios of PM_2.5_/SPM and PM_1.0_/SPM during autumn and winter (0.77 and 0.66) were found compared to spring and summer (0.74 and 0.64). Conversely, in the case of the average ratio of PM_1.0_/PM_2.5_, a slightly higher ratio was found during spring and summer (0.86) compared to autumn and winter (0.85). For example, according to the measurements of the China study [26], the mean ratios of PM_1.0_/PM_2.5_ were 0.71, 0.79, 0.78 and 0.82 in spring, summer, fall and winter, respectively. There were significant differences found among the seasons (*p* < 0.01), and the highest value appeared in winter, similar to other studies [27,28]. When comparing individual filter fractions, a generally higher average ratio of large particles >2.5 μm (filter A) and the smallest particles (UFP, PM_0.25_) <0.25 µm (filter E) was observed in our results during spring and summer, and conversely a higher average ratio of SPM particles in the size range 2.5 to 0.25 µm (filter B, C, D) during autumn and winter (*p* = 0.032). Nevertheless, all mentioned studies indicated in general a strong contribution of small particles in conventional fractions PM_1.0_ and PM_2.5_.

From our results (Table 4, Figure 3), it is evident that the largest part was represented by particles trapped on filter E, i.e., particles with aerodynamic diameters <0.25 µm (PM_0.25_). The mean ratio of these particles represented, on average, 0.43 in the SPM fraction, 0.57 in the PM_2.5_ fraction, and 0.67 in the PM_1.0_ fraction (Table 4). The PM_0.25_ fraction was estimated since no manufacturer offered a personal determination of the fraction defined exactly as PM_0.1_. However, the PM_0.25_ fraction may be evaluated as the PM_0.1_ fraction, due to similarity in the deposition of these ultrafine particles in the pulmonary alveoli [19]. From the ternary diagrams (Figure 3), which show the observed relationships among filter fractions, the predominance of filter E particles in PM_2.5_ (sum of filters B, C, D, E) was evident. Particles of filter fractions C (1.0–0.5 µm) and D (0.5–0.25 µm) demonstrated the lowest deposition efficiency in the alveolar region depending on their spherical particle diameter (d). The deposition efficiency is the amount of particles able to retain and partially accumulate in the respiratory system. On the contrary, for particles of filter fractions E (<0.25 µm) and B (2.5–1.0 µm), the deposition efficiency was growing. Deposition efficiency was the highest around d = 0.01 µm, followed by d = 2.5 µm [19]. The PM_2.5_ fraction, therefore, contained both parts of the deposition. The PM_1.0_ fraction contains mainly one part of the deposition, and PM_0.1_ (in our case PM_0.25_) contains only one part (around d = 0.01 µm). For this reason, PM_1.0_ is determined by continuous monitoring in many places around the world. It is assumed that the part of the respirable fraction expressed in this way associates better with some indicators of health status than PM_2.5_.

In general, the SPM adverse effects on human health are associated with size, sur-face area, and chemical composition, depending on the substances which are bound to the particles, e.g., heavy metals, ions, organic pollutants, microorganisms, nitrates, sulphates or elemental carbon [29]. Several studies have suggested that SPM of smaller size can be more potent in inducing cytotoxic and inflammatory responses in the lung due to their larger surface area to mass ratio [10]. Moreover, SPM of smaller size has potential effects on bioaccumulation, oxidation, and inflammation in the human body [10]. In terms of the genotoxic effects, it is hypothesized that the UFP, due having the highest specific surface area of the SPM [30], is the major carrier of carcinogenic polycyclic aromatic hydrocarbons, which mainly induce particular genotoxic effects [31,32,33].

The adverse effects of the different SPM fractions overlap because the corresponding particle sizes overlap. PM_10_, which includes all finer fractions, has similar effects to finer SPM fractions, although the effects can be distinguished by taking mass into account [34]. PM_10_, PM_2.5_ and PM_1.0_ are measured as mass concentrations, while PM_0.1_ is measured more often as particle number concentration. Particle number concentrations are considered as more suitable parameters because, as the particle size decreases, the number increases, especially when particles approach the size of PM_0.1_. Therefore, not many weight measurements are performed for PM_0.1_ [34]. For example, according to the study of Morawska et al. [35], a typical particle number concentration of PM_0.1_ is 2610 particles/cm^3^ for rural areas and 48,180/cm^3^ for roadsides (10,760/cm^3^ mean global concentration).

However, it is difficult to compare such results with other conventional fractions, and it complicates the determination of individual fraction ratios. For example, the Czech study of Kotlik et al. [36], although it mainly focused on indoor air quality in kindergartens, was one of the few research projects and carried out simultaneous measurements of both mass and particle number concentrations of PM_10_, PM_2.5_, and PM_1.0_. The results showed that the ratios of PM_1.0_/PM_10_, PM_2.5_/PM_10_, PM_1.0_/PM_2.5_ calculated from the particle number concentrations were almost identical (0.978, 0.995 and 0.983) and the mass ratios (0.289, 0.400, 0.650) were completely different in comparison to those from particle number concentrations.

In addition, initial epidemiology studies which analyzed UFP and used particle number concentration for UFP exposure evaluation, did not find consistent relationships with health effects [6]. On the other hand, the results of recent studies, which used UFP mass concentrations pointed to significant associations with premature mortality, and reproductive outcomes (e.g., preterm birth and low birth weight) [37,38,39]. According to these recent studies, UFP mass concentrations can be used for potential UFP exposure evaluation [39,40,41]. These findings can be explained by the fact that UFP may cross cell membranes and are also more available for chemical reactions because they have greater surface area per volume due to the small particle diameter [42]. UFP can deposited deep into the lungs, from which they are not easily removed and, therefore, they can have greater health impacts [8,43].

For example, the air quality standard ISO [19], which specifies sampling conventions for SPM deposition in the human respiratory system, suggests an immediate application of conventions based on mass sampling in health effects research to provide an improved correlation between air quality assessment and observed effects. The particle size range used in this standard is extended below 0.1 µm, where deposition is dominated by diffusion.

Therefore, there is not much data on the proportion of PM_0.1_ in other conventional fractions. Only a few experimental studies have measured PM_0.1_ or a fraction close to PM_1.0_ by their mass [15,16], and our study is one of them. In a study from Italy [15], they also used personal cascade impactor samplers and measured particles <0.25 µm. However, they only stated in the results that particles <0.5 μm accounted for 75% of PM_10_ and particles <0.25 µm formed at least half of that. In our study, we found out that particles <0.5 μm (filter D + E) accounted for 56.4% of SPM and 74.4% of PM_2.5_. In a study from Hanoi [16], PM_0.1_ was sampled using a sampler with an inertial fibrous filter, and PM_2.5_ as well as PM_10_ were collected by a cyclone. In this study, only the ratio PM_0.1_/PM_10_ was calculated, and the result was in the range of 0.06 to 0.1. Our observed ratio, not for PM_0.1_/PM_10_, but for PM_0.25_/SPM, was much higher (0.41). In this study, they found the concentration of PM_0.1_ in the range of 5.36 to 11.9 μg/m^3^ in the wet and dry seasons. In our study, we observed an average concentration of PM_0.25_ 14.5 μg/m^3^ (Table 1).

High concentrations of PM_10_ and PM_2.5_ do not necessarily have to be associated with high concentration of UFPs. High concentrations of PM_0.1_ are, for example, associated with season, low air flow, high humidity, increased number of diesel vehicles, and traffic acceleration after stopping [44]. Although the use of catalytic converters and improvements in engine technology have reduced the SPM concentrations and carbon monoxide from automotive exhaust, the toxicity and number of PM_0.1_ have increased [45,46].

In the second part of our study, we compared the IM with RM (see subsection Procedure to determining the parameters of the impactor measurement). Statistical comparisons of the measurement methods for RM and IM suggests satisfactory agreement of these methods (Figure 4 and Figure 5, Table 6). Similarly, the results of the Italian study [15], which also used personal cascade impactor samplers to measure PM_10_, PM_2.5_, and PM_1.0_, and subsequently compared the results with CEN-EU certified SPM measurements (Zambelli Explorer Plus SPM sampler equipped with proper inertial impactors), demonstrated a good agreement for all considered fractions.

From our analysis of the differences between IM and RM (Table 5), higher differences in measurements was observed only in summer. This was probably due to the fact that low concentrations are measured in summer, often below the detection limit, resulting in lower measurement accuracy. At the same time, the aerosol composition in summer is different than in winter (for example, in winter, there is a great contribution of local heating, etc.). This seasonal trend is obvious even in the most polluted areas. For example, in a study [47] from India, in locations where the mean concentration of PM_2.5_ was near to twice and PM_10_ was almost three times higher than the National Ambient Air Quality standard, higher monthly concentrations in winter were found.

The precision of the IM determination, expressed as a coefficient of variation (respective expanded uncertainty) for individual filters A to D of 12–24% (respectively 25–46%) and filter E of 4% (resp. 9%), is satisfactory for the purposes of epidemiological studies because of the results of SPM fractions—PM_2.5_, PM_1.0_, and PM_0.25_ (on filter E—or PM_0.1_). The higher precision of PM_2.5_ and PM_1.0_ (5% and 10%, respectively) was due to using the sum of mass of individual filters. Compared to the reference method according to EN [24] (LVS: Low Volume Sampling method), IM achieves comparable parameters, because the stated expanded uncertainty of the RM method is 8% and the determined expanded uncertainty of the IM method is 10% The detection limit of the method for sampling 12.96 m^3^ (flow rate 9 l/min in 24 h) was 3.6 µg/m^3^ for A-D filter fraction and 5.1 µg/m^3^ for filter fraction E (PM_0.25_).

## 5. Conclusions

According to our results, the respirable fraction may predominantly consist of UFP. The mean ratios 0.43, 0.57, 0.67 of UFP with an aerodynamic diameter <0.25 μm (PM_0.25_) were found in SPM, PM_2.5_, PM_1.0_. Although a substantial decrease in SPM concentrations over the last two decades has been observed, measures applied to improve air quality are apparently focused on easier removal of larger particles, while the most biologically efficient UFP remain in the ambient air. Measures adjusted to improve air quality should be mainly concerned about these particles. According to many studies, it is considered in general that the smaller the particles, the stronger their biological effects. The smaller particles have higher toxicity due to mechanisms of oxidative stress and inflammation, and the smallest particles (UFP) can be translocated from the lungs to the bloodstream. However, the precise role of UFP in many illnesses is still unknown.

At present, UFP are determined primarily as particle numbers, which complicates comparisons with conventional SPM fractions (determined by mass concentration) and the possible determination of UFP ratio in these fractions. Moreover, the mass concentration used to express ultrafine particle exposure shows more consistent relationships with some health effects, in contrast to using particle number concentration. This study also demonstrated a satisfactory agreement between results from personal samplers used to measure UF and UFP and stationary reference methods.

## Figures and Tables

**Table 1:** SPM fractions and classification of the particles of impactor.

	Particles with Aerodynamic Diameter	Designation
Filter		
A	>2.5 μm	Coarse particles (CP)
B	2.5–1.0 µm	Fine particles (FP)
C	1.0–0.5 µm	
D	0.5–0.25 µm	
E	<0.25 µm	Ultrafine particles (UFP)
SPM fractions		
PM_2.5_	≤2.5 µm	Filters B + C + D + E
PM_1.0_	≤1.0 µm	Filters C + D + E
PM_0.25_	<0.25 µm	Filter E

**Table 2:** Sample characteristics of the original dataset (Q1 = lower quartile, A.M. = arithmetic mean, MED = median, Q3 = upper quartile, IQR = interquartile range).

(n = 75 SAMPLES, 375 Measurements)	Min	Q1	A.M.	MED	Q3	Max	IQR
Filter fraction (µg/m^3^)							
A	<3.6 *	4.428	10.8	6.4	10.7	116.7	6.2
B	<3.6 *	<3.6 *	4.1	<3.6 *	4.7	16.7	2.4
C	<3.6 *	<3.6 *	<3.6 *	<3.6 *	<3.6 *	12.5	2.1
D	<3.6 *	<3.6 *	5.2	3.9	6.9	14.6	4.7
E (PM_0.25_)	2.3	9.4	14.5	11.5	18.0	97.2	8.5
SPM fraction (µg/m^3^)							
SPM (total)	9.1	21.9	37.4	27.5	42.5	256.7	20.6
PM_2.5_	<8.1 *	16.2	26.6	20.9	32.4	140.0	16.2
PM_1.0_	<7.2 *	13.2	22.5	17.2	27.7	123.3	14.5

* Value under detection limit, which was: 3.6 µg/m^3^ for A-D filter fractions; 5.1 µg/m^3^ for filter fraction E (PM_0.25_); 8.1 µg/m^3^ for fraction PM_2.5_; 7.2 µg/m^3^ for fraction PM_1.0_ (see Table 6).

**Table 3:** Transformed variation matrix.

Filter	A	B	C	D	E
A	1.000	0.861	0.762	0.635	0.887
B		1.000	0.927	0.864	0.934
C			1.000	0.959	0.948
D				1.000	0.927
E					1.000

The transformed variation matrix can be interpreted as correlation coefficients: values close to 1 correspond to the low variance of log-ratios of corresponding parts and stable proportionality of those compositions.

**Table 4:** Sample characteristics of the compositions of fractions in SPM, PM_2.5_, PM_1.0_ and * PM_0.25_ (Q1 = lower quartile, C.M = compositional mean, MED = median, Q3 = upper quartile).

	All Seasons						** Spring and Summer						** Autumn and Winter					
Filter	Min	Q1	C.M.	MED	Q3	Max	Min	Q1	C.M.	MED	Q3	Max	Min	Q1	C.M.	MED	Q3	Max
SPM																		
A	0.04	0.17	0.24	0.23	0.32	0.73	0.09	0.18	0.26	0.27	0.37	0.47	0.04	0.15	0.23	0.22	0.28	0.73
B	0.02	0.07	0.11	0.10	0.14	0.33	0.05	0.08	0.10	0.09	0.13	0.23	0.02	0.07	0.11	0.11	0.16	0.33
C	0.02	0.06	0.09	0.08	0.11	0.23	0.04	0.07	0.08	0.08	0.10	0.13	0.02	0.05	0.09	0.08	0.12	0.23
D	0.02	0.08	0.13	0.12	0.19	0.34	0.04	0.07	0.11	0.09	0.13	0.34	0.02	0.09	0.15	0.16	0.20	0.31
* E	0.16	0.35	0.43	0.40	0.46	0.65	0.25	0.40	0.45	0.44	0.46	0.57	0.16	0.29	0.42	0.39	0.46	0.65
PM_2.5_																		
B	0.04	0.10	0.14	0.13	0.21	0.41	0.09	0.11	0.14	0.12	0.16	0.26	0.04	0.09	0.15	0.14	0.22	0.41
C	0.04	0.09	0.11	0.11	0.13	0.26	0.07	0.10	0.11	0.11	0.12	0.16	0.04	0.08	0.11	0.10	0.14	0.26
D	0.07	0.11	0.17	0.16	0.24	0.38	0.07	0.10	0.14	0.12	0.18	0.38	0.07	0.13	0.20	0.21	0.25	0.34
* E	0.32	0.48	0.57	0.56	0.65	0.78	0.41	0.56	0.61	0.61	0.66	0.71	0.32	0.41	0.54	0.52	0.64	0.78
PM_1.0_																		
C	0.04	0.10	0.13	0.13	0.15	0.30	0.08	0.12	0.13	0.13	0.15	0.19	0.04	0.10	0.13	0.12	0.19	0.30
D	0.08	0.13	0.20	0.21	0.29	0.42	0.08	0.12	0.16	0.14	0.21	0.42	0.08	0.16	0.23	0.26	0.30	0.42
E	0.39	0.59	0.67	0.68	0.75	0.84	0.46	0.66	0.71	0.73	0.76	0.84	0.39	0.53	0.64	0.62	0.73	0.84

* PM_0.25_ is the filter E. ** A significant difference between evaluated seasons (spring and summer period to autumn and winter period) was found (*p*-value = 0.032, James test).

**Table 5:** Analysis of differences in measurement methods IM and RM, in total by season.

Differences between IM and RM			
Station A and B	A.M. (95%CI) (µg/m^3^)	*p*-Value ^a^	ICC (95%CI) ^b^
In total (*n* = 36)			
PM_1.0_	1.67 (−0.27; 3.60)	0.090	0.91 (0.83; 0.95)
PM_2.5_	3.17 (0.89; 5.44)	0.008	0.89 (0.80; 0.94)
Summer (*n* = 18)			
PM_1.0_	4.00 (2.24; 5.76)	<0.001	0.17 (−0.30; 0.58)
PM_2.5_	4.94 (2.79; 7.10)	<0.001	0.23 (−0.24; 0.62)
Winter (*n* = 18)			
PM_1.0_	−0.67 (−3.95; 2.61)	0.674	0.88 (0.71; 0.95)
PM_2.5_	1.39 (−2.69; 5.47)	0.483	0.85 (0.64; 0.94)

^a^ *p*-value—the paired *t*-test (the differences were tested for normality assumption with the Shapiro-Wilk’s test). ^b^ ICC = Intraclass Correlation Coefficient. A.M.—arithmetic mean.

**Table 6:** Precision, expanded uncertainty and detection limit.

Fraction (µg/m^3^)	Precision (Mean CV in %)	Expanded Uncertainty (%)	Detection Limit (µg/m^3^)
Filter fraction			
A	12	25	3.6
B	16	31	3.6
C	23	46	3.6
D	14	28	3.6
E (PM_0.25_)	4	9	5.1
SPM fraction			
PM_2.5_	5	10	8.1
PM_1.0_	5	10	7.2

CV = mean coefficient of variance. Extended uncertainty = 2*CV.

## Data Availability

Data are available from the corresponding author upon reasonable request.
